# Specalyzer—an interactive online tool to analyze spectral reflectance measurements

**DOI:** 10.7717/peerj.5031

**Published:** 2018-06-25

**Authors:** Alexander Koc, Tina Henriksson, Aakash Chawade

**Affiliations:** 1 Department of Plant Breeding, Swedish University of Agricultural Sciences, Alnarp, Sweden; 2 Lantmännen Lantbruk, Svalöv, Sweden

**Keywords:** Specalyzer, Spectroradiometer, Proximal phenotyping, Online tool, Spectral reflectance, Vegetation indices

## Abstract

Low-cost phenotyping using proximal sensors is increasingly becoming popular in plant breeding. As these techniques generate a large amount of data, analysis pipelines that do not require expertise in computer programming can benefit a broader user base. In this work, a new online tool Specalyzer is presented that allows interactive analysis of the spectral reflectance data generated by proximal spectroradiometers. Specalyzer can be operated from any web browser allowing data uploading, analysis, interactive plots and exporting by point and click using a simple graphical user interface. Specalyzer is evaluated with case study data from a winter wheat fertilizer trial with two fertilizer treatments. Specalyzer can be accessed online at http://www.specalyzer.org.

## Background

High-throughput plant phenotyping (HTPP) is becoming increasingly popular with the development of new low-cost phenotyping technologies and sensors. HTPP can aid in the detection of plant traits for applications in breeding and farming and for gaining fundamental understanding of molecular mechanisms underlying the trait of interest (Furbank & Tester, 2011). HTPP can be performed on individual plants, trial plots or big farms. Sensors are available for estimating spectral reflectance in the leaves of individual plants or small plots to large scale phenotyping of big farms with unmanned aerial vehicles mounted with hyperspectral cameras or with satellite imaging (Muñoz Huerta et al., 2013; Tattaris, Reynolds & Chapman, 2016).

Phenotyping by proximal spectroradiometers can be performed to estimate various traits in several different crops. Canopy biomass and nitrogen status in wheat was estimated with a proximal spectrometer with a wavelength range of 400–900 nm mounted on a tractor (Hansen & Schjoerring, 2003), leaf area index in rice was measured with a handheld spectrometer with a wavelength range of 250–2,500 nm (Wang et al., 2007), nitrogen uptake in winter wheat was estimated with a handheld spectrometer with a wavelength range of 350–1,000 nm (Yao et al., 2013), grain yield and protein content in winter wheat was also measured with a handheld spectroradiometer with a wavelength range of 447–1,752 nm (Xue, Cao & Yang, 2007) and wheat yield under irrigation was estimated with a portable spectroradiometer with a wavelength range of 350–1,100 nm (Babar et al., 2006).

A large number of vegetation indices (VIs) have been developed using the visible and near-infrared spectral wavelengths for estimation of various traits of interest in plants (Agapiou, Hadjimitsis & Alexakis, 2012). These VIs can be estimated from the spectral reflectance from the proximal spectroradiometers (Deery et al., 2014; Gizaw, Garland-Campbell & Carter, 2016; Hansen & Schjoerring, 2003), and with the sensors from unmanned aerial vehicles and satellites (Franke & Menz, 2007; Haghighattalab et al., 2016; Tattaris, Reynolds & Chapman, 2016; Tucker & Sellers, 1986). As the VIs and their association with a trait of interest is often known, the VIs estimated from the new measurements can aid in detection of the associated traits. VIs have proven to be effective in estimation of leaf area index (Tucker & Sellers, 1986; Wang et al., 2007), radiation use efficiency (Peñuelas, Filella & Gamon, 1995), water status (Peñuelas et al., 1993), leaf pigments (Sims & Gamon, 2002), grain yield (Babar et al., 2006; Cao et al., 2015; Gizaw, Garland-Campbell & Carter, 2016; Xue, Cao & Yang, 2007) and diseases (Cao et al., 2015; Mahlein, 2016; Odilbekov et al., 2018). VIs have also been used in conjunction with multivariate and machine learning techniques to build advanced models for improved detection of complex traits (Mahlein, 2016; Odilbekov et al., 2018; Singh et al., 2016). VIs have been most successfully used for efficient application of fertilizers in the field. Leaching of fertilizer leads to ground and water pollution and wastage of resources, and thus, improving the nitrogen use efficiency of the crops and the efficient application of fertilizers is important for sustainable agriculture (Chawade et al., 2018; Muñoz Huerta et al., 2013).

Spectral data has to be processed upon acquisition and the steps involve pre-processing to remove outlier samples, normalization, trimming of edges to remove low signal-to-noise ratio wavelengths and estimation of VIs. The R packages hsdar (Lehnert, Meyer & B, 2017) and pavo (Maia et al., 2013) provide most of the features required for analysis of the collected spectral data in the R command line environment. The package pavo additionally provides features for visualization of the data. While the two packages are efficient in analyzing the data through a command line interface, they lack a graphical user interface and require users to be familiar with the R environment. This can be challenging for users unfamiliar with computer programming. The target users of spectral data analysis are primarily biologists and plant breeders who are interested in studying the trait of interest using spectral reflectance. Thus, a tool with a graphical user interface for spectra data analysis will allow a broader user base to work with such data.

Analysis tools with graphical user interface independent of the operating system enable ease of use and a broader acceptance of spectral reflectance techniques. Visualization tools allow users with little or no skills in computer programming to analyze multidimensional phenotypic data and identify dominant patterns relevant to their research question. Two aspects are of fundamental importance for building a visualization tool, namely, data analysis capabilities and user experience (UX). Data readability and interpretability can be improved with graphical representation with several different types of plots including boxplots, barplots, heatmaps, histograms and scatter plots (Calatroni & Wildfire, 2017; Chawade, Alexandersson & Levander, 2014). Appropriate selection of colors and sizes of various data points in the plots connects the graphic to the real world (Yau, 2013b). Although automation of data analysis is desired for high-throughput experiments, a balance between automation and visualization based decision making is at times necessary. The field of visual analytics allows the achievement of this goal and is the subject of ongoing research (O’Donoghue et al., 2010). UX aspects necessitate developing a user friendly graphical interface (Pavelin et al., 2012) which could be simplistic and web-based (Chawade, Alexandersson & Levander, 2014) or require a local installation offering advanced customization possibilities (Kerren et al., 2017; Shannon, 2003).

In this work, a new online tool Specalyzer is proposed which enables analysis and visualization of the collected spectral data in a web browser and thus can be used on any device with a web browser and an internet connection. Various features in Specalyzer are described and evaluated with a case study on fertilizer treatment in winter wheat.

## Methods and Materials

### Implementation

Specalyzer is a web application implemented in the statistical programming language R v3.4.2 (R Development Core Team, 2016) and built using the Shiny v1.0.5 web application framework (Chang et al., 2017). Specalyzer uses the the hsdar v0.5.1 package (Lehnert, Meyer & B, 2017) and the included Speclib function for managing spectral data and calculating VIs. Plotly v4.7.1 (Sievert et al., 2017) is used to generate interactive visualizations shown in the web application. Specalyzer also uses dplyr v0.7.4 (Wickham et al., 2017a) and reshape2 v1.4.2 (Wickham, 2007) for extracting and transforming data, and asdreader v0.1-3 (Roudier, 2017) for reading binary ASD FieldSpec^®^ data files. Together, these packages are used for data input, processing, and visualization functionality for spectral data. The package shinyjs v1.0 (Attali, 2018) is used for additional user-interface functionality, and readr v1.1.1 (Wickham, Hester & Francois, 2017b) for reading data from disk and generating tables for export. Specalyzer code is available at https://github.com/alkc/specalyzer.

### Data input

The spectral data formats supported by Specalyzer are (a) ASD FieldSpec^®^ binary spectral data files, (b) SpectraWiz^®^ data files and (c) data merged in a single generic text file. Continuous and/or discrete attribute data in the form of a tab-delimited table can also be uploaded. The attribute data should be organized so that each column is a trait and each of the rows correspond to a spectral sample. The first column should be labelled “filename” in the header row and should include the filenames of the corresponding spectral data files. Additionally, a comma-delimited matrix of filenames can be uploaded that includes the spatial distribution of the samples in the field. The spatial matrix is used for visualization of the VI or attributes of samples in the field.

### Estimation of vegetation indices

Specalyzer estimates 140 VIs within the spectral range of 400–1,000 nm and are summarized in File S1. If the reflectance measurements of the wavelengths required for estimating a VI is unavailable, missing values are reported for the given index. Thus, to estimate all indices provided by Specalyzer, data acquisition with spectroradiometers with a resolution of one nanometer is recommended. Specalyzer does not perform any data interpolation and thus such data transformation can be necessary when the data is collected with spectral instruments with lower resolutions. Such data transformation should be performed prior to using Specalyzer.

### Case study data

A winter wheat field trial was conducted in Svalöv, in Southern Sweden in 2015–2016 with two fertilizer treatments (140 and 180 kg ha^−1^). The spectral reflectance from 10 breeding lines was measured with the handheld Apogee PS-100 spectroradiometer (Apogee Instruments Inc., Logan, UT, USA). The spectroradiometer was calibrated against the white reference at every tenth reading and the measurements were made in the range of 339–1,100 nm. Due to a low signal-to-noise ratio in the areas around the edges of the measured spectral interval, the data in the range of 400–1,000 nm was considered for further analysis. The measurements were made in June 2016 around midday under a clear sky at the post-anthesis growth stage (Zadoks 71–77). The spectroradiometer was held approximately 1 m above the canopies for reflectance measurements.

## Results

### Features available in Specalyzer

**Figure 1:**
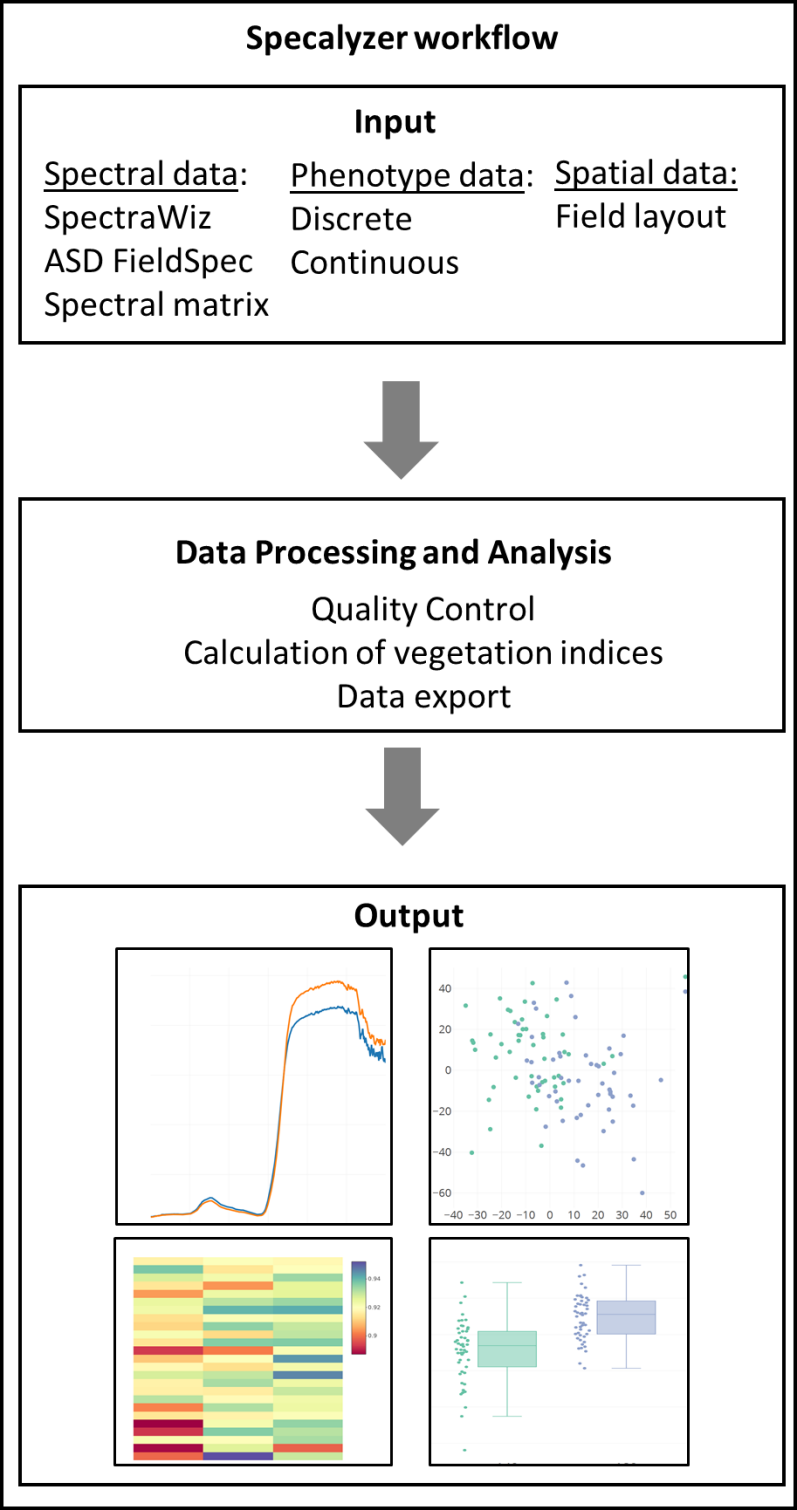
Specalyzer workflow. Specalyzer workflow for data input, different aspects of data processing and analysis, and output visualization.

**Figure 2:**
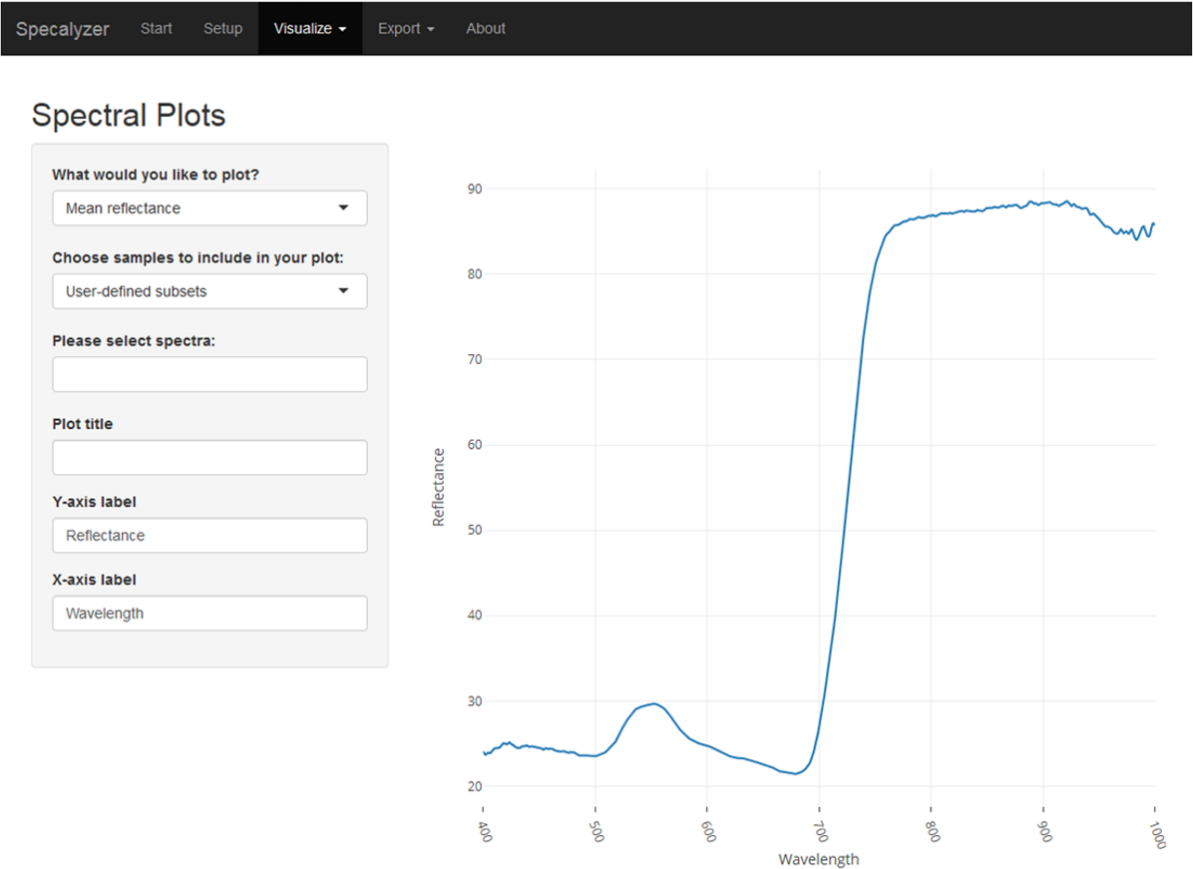
Specalyzer web application screenshot. A screenshot of the Specalyzer web-application displaying the menu for generating spectral plots from spectral reflectance data.

The aim of Specalyzer is to aid in the quality control, pre-processing, estimation of VIs and visualization of the spectral reflectance data (Fig. 1). This is achieved with a web application with an interactive user interface capable of processing and visualizing raw and processed data (Fig. 2). Specalyzer is platform independent and can be used in a web browser on any computer or mobile device. The available pre-processing features are removing outlier samples, trimming the spectral range and calculation of 140 VIs. Information about the replicates can be optionally included in the attribute file and various plots can be created by grouping the samples by the provided attribute(s). VIs relevant to a trait of interest can be identified by performing correlation analysis (quantitative trait) or one-way ANOVA analysis (quantitative trait).

The available plotting features for the spectral data are scatterplots for individual samples, mean and median of all samples, and the variance component. For the VIs, boxplots can be created for ordinal attributes and scatterplots for continuous attributes. Optionally, the data points (samples) can be overlaid over the boxplots. Additionally, a custom VI can also be manually added to the list of VIs. Samples can be grouped by any provided attribute for further analysis and plotting. PCA plots can be created from the spectral data to analyze sample grouping, data structure and outliers. In the PCA plot, automatic sample outlier detection is available and is based on standard deviation of a sample from the mean of the loadings of principal components 1 and 2. Fieldmaps can be created to visualize the spatial distribution of samples in the field and the spatial variation in the intensities of a given VI. All plots in Specalyzer are interactive providing further information on each datapoint in the plot by hovering the mouse pointer over it. Finally, various plots can be saved in portable network graphics (PNG) format and the spectral and the VI data can be exported for further analysis. While exporting, samples can also be averaged based on any provided attributes.

### Evaluation of Specalyzer

The features in Specalyzer were evaluated in a case study from a fertilizer field trial with two treatments of fertilizer levels.

### Case study

**Figure 3:**
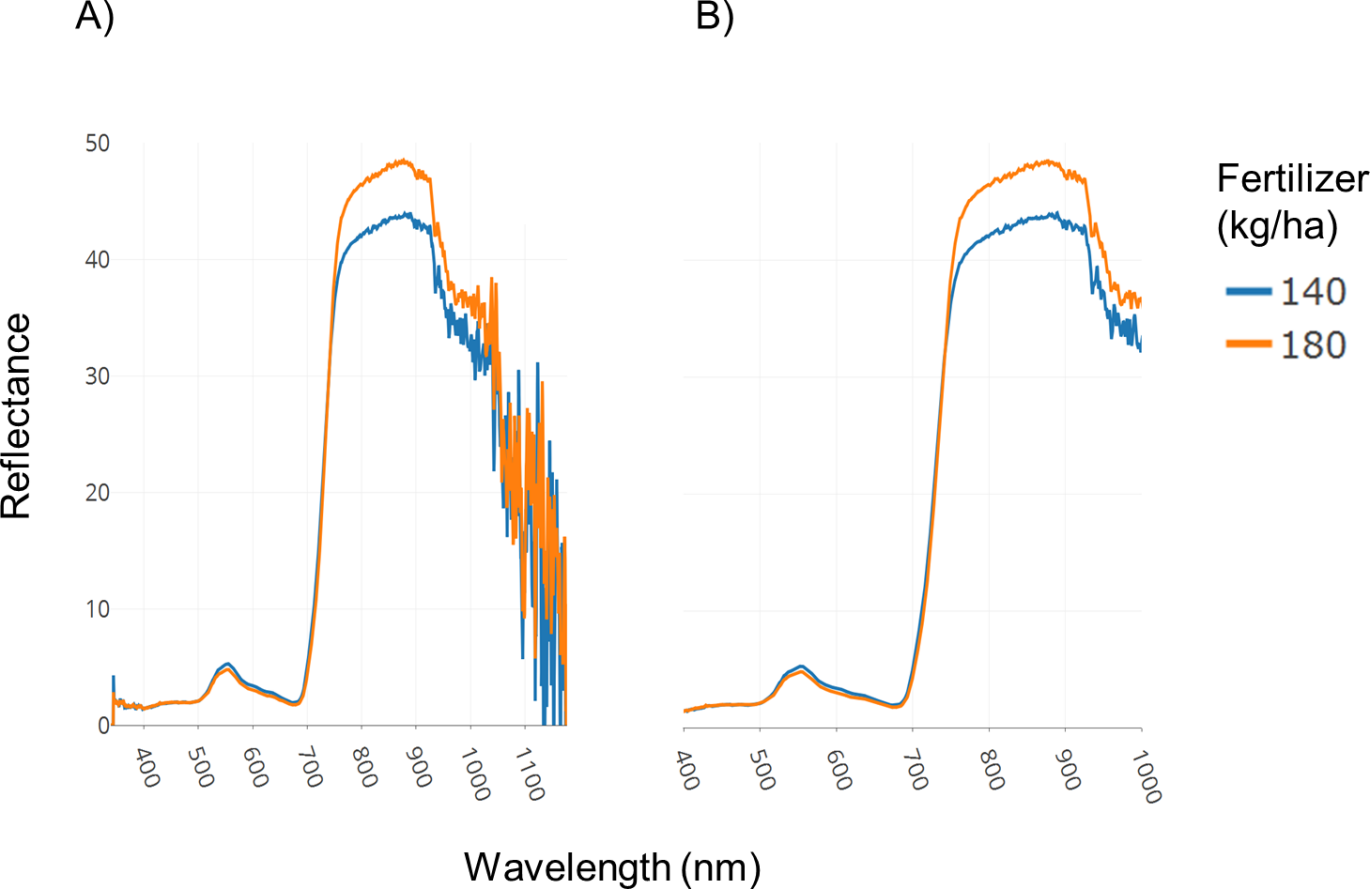
Filtering regions with low signal-to-noise ratio. Demonstration of the effect of removing regions with low signal-to-noise ratio in spectral data within Specalyzer. (A) Aggregated spectral data showing low signal-to-noise ratio in the intervals 300–400 and 1,000–1,200 nm (B) Aggregated spectral data after trimming away noisy regions.

**Figure 4:**
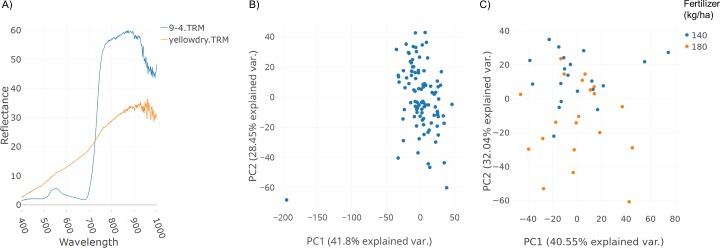
Outlier detection in Specalyzer. Outlier detection and removal in spectral data using Specalyzer. (A) Reflectance data collected from a winter wheat canopy (blue) compared to reflectance data collected from dry leaves (orange) (B) PCA clustering analysis of the same spectral data set with spectral samples from several wheat canopies clustering to the right and the dry leaf sample appearing to the left as an outlier (C) Recalculated PCA plot with outlier dry leaf spectral sample removed, and with the remaining wheat canopy spectral samples colored by fertilizer treatment (140 and 180 kg ha^−1^).

A field trial was conducted with replications and two fertilizer treatments (140 and 180 kg ha^−1^). Spectral data was collected as described in the Methods section. Figure 3A illustrates spectral plots from Specalyzer where regions between 300–400 nm and 1,000–1,200 nm have low signal-to-noise ratio. These regions can be filtered away by masking unwanted regions and new plots can be generated for further analysis (Fig. 3B). Another important quality control (QC) feature in Specalyzer is outlier detection with PCA plots (Fig. 4). For the case study data, an outlier is detected in the PCA plot (Fig. 4B), and the sample label is identified by hovering the mouse over the outlier sample. A spectral plot with the outlier sample together with another randomly chosen sample shows drastically different spectral reflectance profiles (Fig. 4A). The outlier sample in this case study was a control sample of dry leaves. The new PCA plot after filtering away the outlier sample shows uniform distribution of samples (Fig. 4C).

The plots shown in Figs. 3 and 4 can also be used to compare samples by attributes for investigating the spectral reflectance in response to different attributes. For example, in the case study, the spectral plots in Fig. 3B shows the difference in mean reflectance in samples from two fertilizer treatments. The mean reflectance of samples vary for the two treatments with the samples receiving more fertilizer showing increased reflectance in the near-infrared spectrum. Samples can also be colored by attributes in the PCA plot (Fig. 4C).

**Figure 5:**
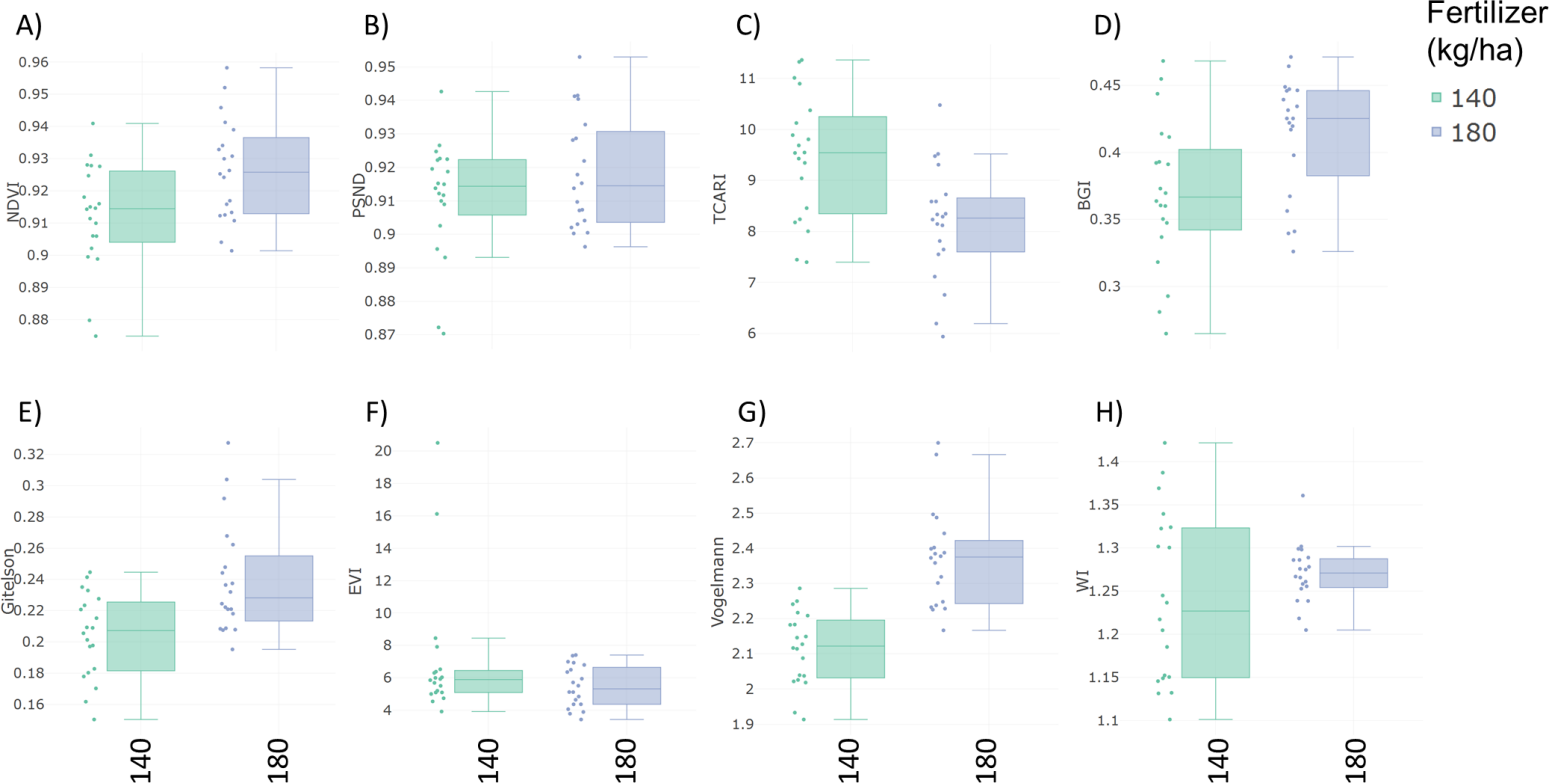
Boxplots of vegetation indices. Vegetation index boxplots from spectral data collected from winter wheat canopies aggregated by two fertilizer treatments (140 and 180 kg ha^−1^). (A) Normalized Difference Vegetation Index (NDVI) (B) Pigment Specific Normalized Difference (PSND) (C) Transformed Chlorophyll Absorption Reflectance Index (TCARI) (D) BGI (E) Gitelson Index (F) Enhanced Vegetation Index (EVI) (G) Vogelmann index (H) Water Index (WI).

**Figure 6:**
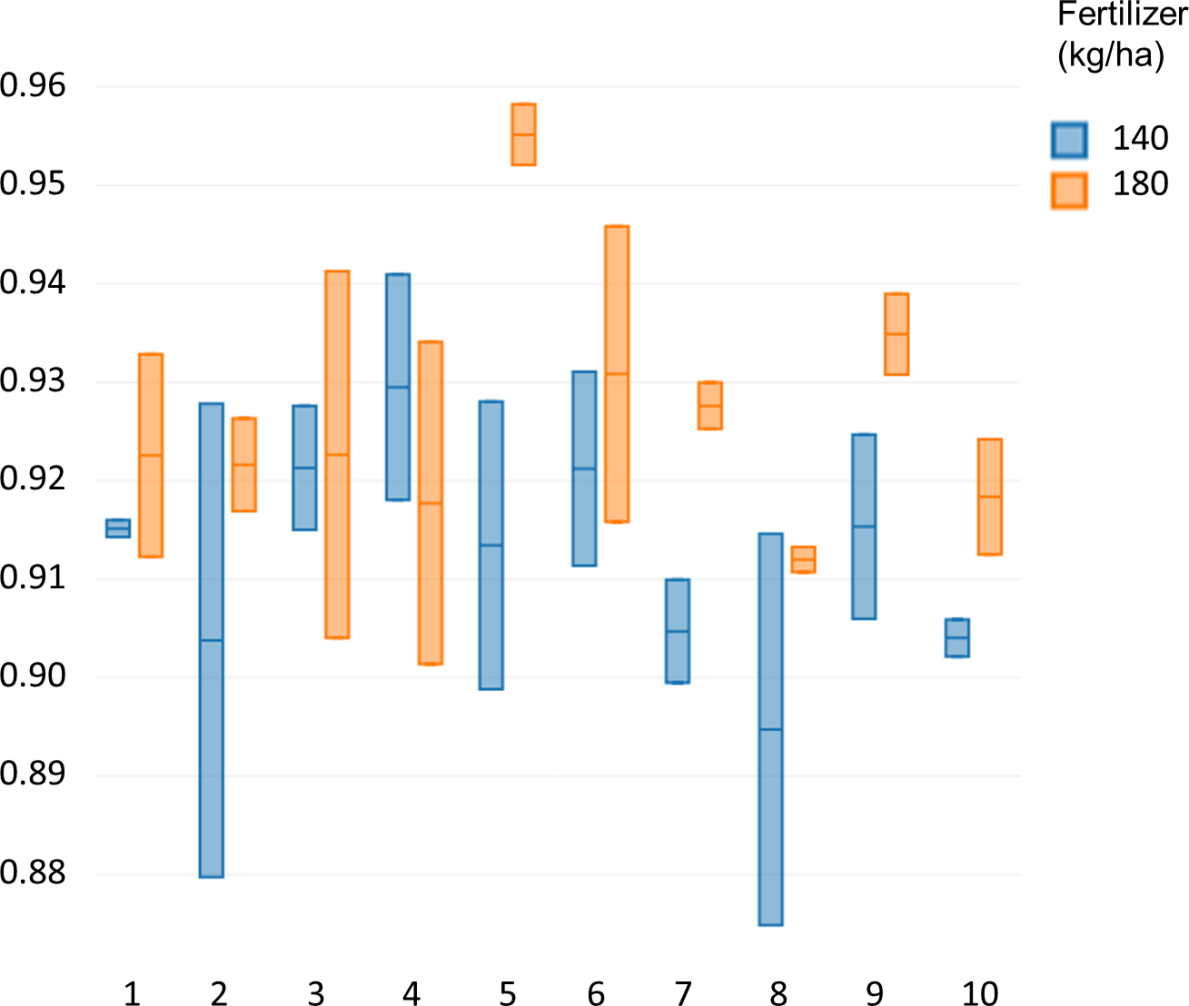
NDVI of the winter wheat cultivars. Comparison of NDVI values between 10 different winter wheat cultivars for two fertilizer treatments (140 and 180 kg ha^−1^).

**Figure 7:**
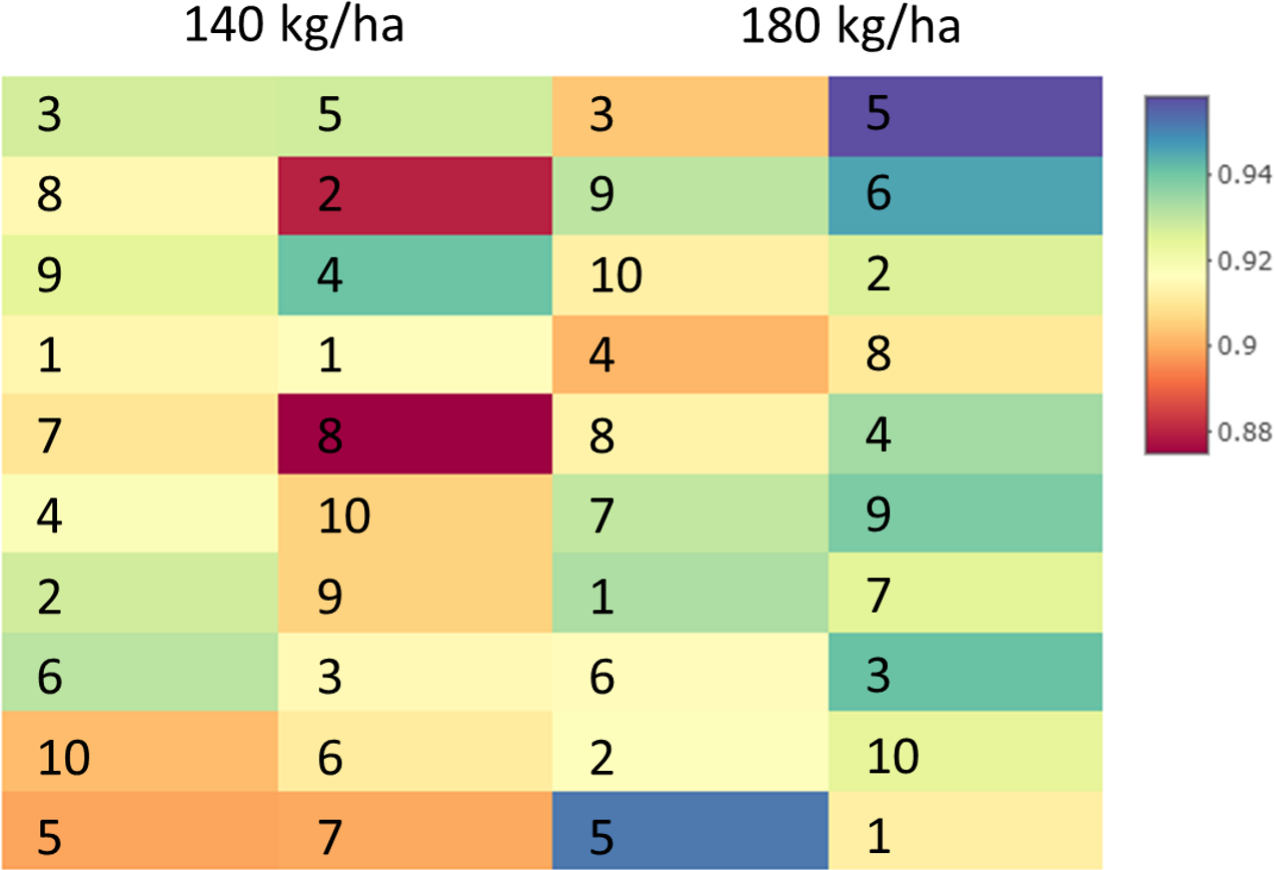
Fieldmap with NDVI. NDVI intensities visualized as a spatial grid corresponding to plots in a field trial. Each rectangle corresponds to an NDVI intensity from a specific wheat cultivar plot in a field trial where the cultivars were subjected to two different fertilizer treatments (140 and 180 kg fertilizer ha^−1^).

Specalyzer also calculates 140 previously known VIs from the spectral data. These indices can be calculated for individual spectral samples, or can be aggregated by attribute for scatterplots and boxplots (Fig. 5). Boxplots for a few selected indices are shown for the case study data where treatment differences are observed for the indices such as NDVI and TCARI while no differences in the treatment can be seen for the indices EVI and WI (Fig. 5). Boxplots can also be created for individual samples or for sample groupings. In a boxplot with samples grouped by replication, variation in NDVI can be seen in the breeding lines with breeding line 5 having the highest NDVI in the treatment group with 180 kg fertilizer ha^−1^ (Fig. 6). Similar plots can be created for over 140 VIs allowing detailed analysis of the VIs and the treatments. Specalyzer also supports visualizing indices against continuous traits in scatterplots. Furthermore, fieldmaps can be created to visualize the spatial distribution of the measurements in the field and the corresponding intensities of the indices. In Fig. 7, the sample number 5 can be identified with higher NDVI levels in the treatment group with 180 kg fertilizer ha^−1^ and from the fieldmap it can be seen that higher NDVI is specific for sample 5 indicating that this sample might have higher nutrient use efficiency. The processed spectral and the VI data can be exported for further analysis in a statistical software.

## Discussion

Effectiveness of data visualization can be estimated based on user interactivity, ability to integrate data from different sources and ease of access to the tool (Yau, 2013a; Zhu, Hoon & Teow, 2015). Visualization allows ease of use and greater insight into the data in a way that is not obvious from descriptive statistics (Calatroni & Wildfire, 2017). Interactivity in data visualization facilitates overview of data followed by zooming and filtering the plots when required for greater details (Shneiderman, 1996). The graphical user interface of Specalyzer makes it an easy-to-use web application for exploring spectral reflectance, attributes and spatial information of datasets in any web browser, without requiring programming skills or having to install a software. The data visualization functions in Specalyzer are useful for both QC and for analyzing spectral data in relation to the attribute data. The plots in Specalyzer are interactive, allowing users to explore the data interactively to identify dominant patterns, new insights and decision making for the underlying traits of interest. Specalyzer currently accepts data from two spectroradiometer vendors, ASD FieldSpec^®^ (Malvern Panalytical, Malvern, Worcestershire, UK) and Apogee SpectraWiz^®^ (Apogee Instruments Inc., Logan, UT, USA) format. Additionally, it also accepts tabulated data in a generic text file format. This allows broader application of Specalyzer for data obtained from various spectroradiometer devices.

Large data sets have hundreds of variables increasing the data complexity and thus making them difficult to visualize. Dimensionality reduction methods such as PCA allow visualizing data in a two-dimensional plane where samples with similar profiles are clustered closer together while dissimilar samples are separated in space which allows visualization of the clustering patterns and the underlying similarity matrices (Calatroni & Wildfire, 2017). In Specalyzer, the interactive PCA plot has the features to zoom and modify color and size of the data points based on user provided attributes, enabling analysis of trait of interest and detecting outliers.

Specalyzer is implemented as an online tool and thus can be used with any mobile platform with a web browser and access to internet. It is built with the R programming language with an interactive graphical user interface using the Shiny web application. The zooming, panning and tooltips features in charts are provided by plotly R package which is a high-level interface to the JavaScript plotting library plotly.js. This allows interactive data analysis where the output is continuously updated based on changes to the parameters by the user. There are several advantages to using Shiny and plotly in Specalyzer, (a) User-friendliness for analyzing big datasets; (b) Platform independence allowing flexibility in using devices; (c) Customized charts allowing greater control; (d) Interactivity to easily identify outliers and data points of interest and (e) Publication-quality figures. This enables, for example, analysis of the collected data with a mobile phone while in the field which can facilitate identifying individual plots using Specalyzer for further manual inspection in the field. This can save time for germplasm evaluation in the field thus reducing costs. Phenotyping carts are mounted with proximal sensors such as RGB and hyperspectral cameras, infrared thermometers and spectroradiometers which are being developed and are operated from a computer (Deery et al., 2014). Specalyzer can be further modified to be used with spectroradiometers on these carts enabling instantaneous analysis of the acquired data in the field.

Future work on Specalyzer will involve expanding the data visualization toolkit and improving existing data visualization functionality. For example, an important improvement is to enable users to aggregate spectral measurements by more than one attribute. Another important improvement in the data visualization menus would be adding plot layout and output controls for users to get customized publication-ready figures out of the application. Currently, VIs estimated by Specalyzer can be exported for further analysis. In a parallel project on wheat, we estimated VIs in Specalyzer and thereafter using machine learning, identified key VIs to detect the fungal disease Septoria tritici blotch of wheat (Odilbekov et al., 2018). Thus, another beneficial feature in Specalyzer would be to include various machine learning methods to classify samples and identify key VIs underlying a trait of interest. Based on the case study presented here and the previous work (Odilbekov et al., 2018) we suggest that Specalyzer can be a useful tool for analyzing spectral reflectance data.

## Conclusion

Efficient management and analysis of the phenotypic data is crucial and thus there is a clear need for development of new tools that allow users with broader expertise to analyze and interpret the acquired data. As this work demonstrated, Specalyzer provides an interactive graphical user interface for spectral data analysis and for estimation of several previously known VIs. Analyzing big datasets is a challenging task and thus Specalyzer can help facilitate this process. Further work is required to introduce additional features such as machine learning for variable selection and spatial analysis.

##  Supplemental Information

10.7717/peerj.5031/supp-1 File S1 List of vegetation indices used in this work

## Additional Information and Declarations

### Data Availability

The following information was supplied regarding data availability: The data used in this manuscript is available from the the software webpage http://www.specalyzer.org. Specalyzer code can be accessed at https://github.com/alkc/specalyzer.
