# Supplementary material for: Specalyzer—an interactive online tool to analyze spectral reflectance measurements
Source: PeerJ. 2018 Jun 25;6:e5031. doi: 10.7717/peerj.5031 (PMC6022728; doi:10.7717/peerj.5031)
Supplement: File S1 [file peerj-06-5031-s001.pdf]

Supplementary File 1: List of vegetation indices used in this work.

Vegetation indices used in the study (adapted from (Agapiou, Hadjimitsis, & Alexakis, 2012; Lehnert, Meyer, & Bendix, 2016; Main et al., 2011; Odilbekov, Armoniené, Henriksson, & Chawade, 2018))

| No | Abbr | Name | Formula | Reference |
|---|---|---|---|---|
| 1 | ARI | Anthocyanin Reflectance Index | $(1/R_{550}) - (1/R_{700})$ | (A. A. Gitelson, Merzlyak, & Chivkunova, 2001) |
| 2 | ARI2 | Anthocyanin Reflectance index 2 | $R_{800}(1/R_{550}) - (1/R_{700})$ | (A. A. Gitelson et al., 2001) |
| 3 | BGI | Blue Green Pigment Index | $R_{450}/R_{550}$ | (P. Zarco-Tejada et al., 2005) |
| 4 | SB703/Boochs | Single Band 703 Boochs | $D_{703}$ | (Boochs, Kupfer, Dockter, & Kühbauch, 1990) |
| 5 | SB720/Boochs2 | Single Band 720 Boochs 2 | $D_{720}$ | (Boochs et al., 1990) |
| 6 | BRI | Browning Reflectance Index | $R_{450}/R_{690}$ | (P. Zarco-Tejada et al., 2005) |
| 7 | CAI | Cellulose Absorption Index | $0.5 * (R_{2000} + R_{2200}) – R_{2100}$ | (Nagler, Inoue, Glenn, Russ, & Daughtry, 2003) |
| 8 | CARI | Chlorophyll Absorption Ratio Index | $R_{700} * abs(a * 670 + R_{670} + b)/R_{670} * (\alpha^2 + 1)^{0.5}$<br>$\alpha = (R_{700} - R_{550})/150$<br>$b = R_{550} - (550 * \alpha)$ | (Kim, Daughtry, Chappelle, McMurtrey, & Walthall, 1994) |
| 9 | Ctr | Carter | $R_{695}/R_{420}$ | (Carter, 1994) |
| 10 | Ctr2 | Carter 2 | $R_{695}/R_{760}$ | (Carter, 1994) |
| 11 | Ctr3 | Carter 3 | $R_{605}/R_{760}$ | (Carter, 1994) |
| 12 | Ctr4 | Carter 4 | $R_{710}/R_{760}$ | (Carter, 1994) |
| 13 | Ctr5 | Carter 5 | $R_{695}/R_{670}$ | (Carter, 1994) |
| 14 | Ctr6 | Carter 6 | $R_{550}$ | (Carter, 1994) |

| | | | | |
|---|---|---|---|---|
| 15 | CI | Coloration Index | $R_{675} * R_{690}/R^2_{683}$ | (P. J. Zarco-Tejada, Pushnik, Dobrowski, & Ustin, 2003) |
| 16 | CI2 | Coloration Index 2 | $R_{760}/R_{700} - 1$ | (A. A. Gitelson, Gritz, & Merzlyak, 2003) |
| 17 | ClAInt | - | $\int_{600nm}^{735nm} R$ | (Oppelt & Mauser, 2004) |
| 18 | CRI | Carotenoid Reflectance Index | $(1/R_{510}) - (1/R_{550})$ | (A. A. Gitelson, Zur, Chivkunova, & Merzlyak, 2002) |
| 19 | CRI1 | Carotenoid Reflectance Index 1 | $1/R_{515} - 1/R_{550}$ | (A. A. Gitelson et al., 2003) |
| 20 | CRI2 | Carotenoid Reflectance Index 2 | $1/R_{515} - 1/R_{770}$ | (A. A. Gitelson et al., 2003) |
| 21 | CRI3 | Carotenoid Reflectance Index 3 | $1/R_{515} - 1/R_{550} * R_{770}$ | (A. A. Gitelson et al., 2003) |
| 22 | CRI4 | Carotenoid Reflectance Index 4 | $1/R_{515} - 1/R_{700} * R_{770}$ | (A. A. Gitelson et al., 2003) |
| 23 | D1 | Derivative index | $D_{730}/D_{706}$ | (P. J. Zarco-Tejada, Pushnik, et al., 2003) |
| 24 | D2 | Derivative index | $D_{705}/D_{722}$ | (P. J. Zarco-Tejada, Pushnik, et al., 2003) |
| 25 | Datt | Datt | $(R_{850} - R_{710})/(R_{850} - R_{680})$ | (B. Datt, 1999) |
| 26 | Datt2 | Datt 2 | $R_{850}/R_{710}$ | (B. Datt, 1999) |
| 27 | Datt3 | Datt 3 | $D_{754}/D_{704}$ | (B. Datt, 1999) |
| 28 | Datt4 | Datt 4 | $R_{672}/(R_{550} * R_{708})$ | (Bisun Datt, 1998) |
| 29 | Datt5 | Datt 5 | $R_{672}/R_{550}$ | (Bisun Datt, 1998) |
| 30 | Datt6 | Datt 6 | $R_{860}/(R_{550} * R_{708})$ | (Bisun Datt, 1998) |
| 31 | Datt7 | Datt 7 | $(R_{860} - R_{2218})/(R_{860} - R_{1928})$ | |
| 32 | Datt8 | Datt 8 | $(R_{860} - R_{1788})/(R_{860} - R_{1928})$ | |
| 33 | DD | Double Difference Index | $(R_{749} - R_{720}) - (R_{701} - R_{672})$ | (le Maire, François, & Dufrêne, 2004) |

| | | | | |
|---|---|---|---|---|
| 34 | DDn | New Double Difference Index | $2 * (R_{710} - R_{660} - R_{760})$ | (le Maire et al., 2008) |
| 35 | DPI | Double Peak Index | $(D_{688} - D_{710})/D^2_{697}$ | (P. J. Zarco-Tejada, Pushnik, et al., 2003) |
| 36 | DWSI1 | Disease water stress index 1 | $R_{800}/R_{1660}$ | (Apan, Held, Phinn, & Markley, 2004) |
| 37 | DWSI2 | Disease water stress index 2 | $R_{1660}/R_{550}$ | (Apan et al., 2004) |
| 38 | DWSI3 | Disease water stress index 3 | $R_{1660}/R_{680}$ | (Apan et al., 2004) |
| 39 | DWSI4 | Disease water stress index 4 | $R_{550}/R_{680}$ | (Apan et al., 2004) |
| 40 | DWSI5 | Disease water stress index 5 | $(R_{800} + R_{550})/(R_{1660} + R_{680})$ | (Apan et al., 2004) |
| 41 | EGFN | Edge green first derivative normalized difference | $(max(D_{650:750}) - max(D_{500:550}))/ (max(D_{650:750}) + max(D_{500:550}))$ | (Peñuelas, Gamon, Fredeen, Merino, & Field, 1994) |
| 42 | EGFR | Edge green first derivative ratio | $max(D_{650:750})/ max(D_{500:550})$ | (Peñuelas et al., 1994) |
| 43 | EVI | Enhanced Vegetation Index | $2.5 * ((R_{800} - R_{670})/(R_{800} - (6 * R_{670}) - (7.5 * R_{475}) + 1))$ | (A.R. Huete, Liu, Batchily, & Van Leeuwen, 1997) |
| 44 | GDVI2 | Green Difference Vegetation Index 2 | $(R^2_{800} - R^2_{680})/(R^2_{800} + R^2_{680})$ | (W. Wu, 2014) |
| 45 | GDVI3 | Green Difference Vegetation Index 3 | $(R^3_{800} - R^3_{680})/(R^3_{800} + R^3_{680})$ | (W. Wu, 2014) |
| 46 | GDVI4 | Green Difference Vegetation Index 4 | $(R^4_{800} - R^4_{680})/(R^4_{800} + R^4_{680})$ | (W. Wu, 2014) |
| 47 | GI | Greenness Index | $R_{554}/R_{677}$ | (Smith, Adams, Stephens, & Hick, 1995) |
| 48 | - | Gitelson | $1/R_{700}$ | (A. A. Gitelson, Buschmann, & Lichtenthaler, 1999) |
| 49 | - | Gitelson 2 | $(R_{750} - R_{800})/(R_{695} - R_{740}) - 1$ | (A. A. Gitelson et al., 2003) |
| 50 | GMI1 | Gitelson and Merzlyak Index 1 | $R_{750}/R_{550}$ | (A. A. Gitelson & Merzlyak, 1998) |
| 51 | GMI2 | Gitelson and Merzlyak Index 2 | $R_{750}/R_{700}$ | (A. A. Gitelson & Merzlyak, 1998) |
| 52 | Green NDVI | Green Normalized Difference Vegetation Index | $(R_{800} - R_{550})/(R_{800} + R_{550})$ | (A. A. Gitelson, Kaufman, & Merzlyak, 1996) |

| | | | | |
|---|---|---|---|---|
| 53 | GVI | Greenness Vegetation Index | $(R_{682} - R_{553})/(R_{682} + R_{553})$ | (Gandia, Fernández, García, & Moreno, 2004) |
| 54 | LIC | Lichtenthaler indices | $R_{440}/R_{690}$ | (Lichtenthaler, Lang, Sowinska, Heisel, & Miehé, 1996) |
| 55 | LRDSI1 | Leaf Rust Disease Severity Index 1 | $6.9*(R_{605}/R_{455})-1.2$ | (Ashourloo, Mobasheri, & Huete, 2014) |
| 56 | LRDSI2 | Leaf Rust Disease Severity Index 2 | $4.2 * (R_{695}/R_{455}) - 0.38$ | (Ashourloo et al., 2014) |
| 57 | LWVI1 | Normalized Difference 1094/983 Leaf water VI 1 | $(R_{1094} - R_{983}) / (R_{1094} + R_{983})$ | (Galvão, Formaggio, & Tisot, 2005) |
| 58 | LWVI2 | Normalized Difference 1094/1205 Leaf water VI 2 | $(R_{1094} - R_{1205}) / (R_{1094} + R_{1205})$ | (Galvão et al., 2005) |
| 59 | - | Maccioni | $(R_{780} - R_{710})/(R_{780} - R_{680})$ | (Maccioni, Agati, & Mazzinghi, 2001) |
| 60 | MCARI | Modified Chlorophyll Absorption in Reflectance Index | $((R_{700} - R_{670}) - 0.2 * (R_{700} - R_{550})) * (R_{700}/R_{670})$ | (Daughtry, Walthall, Kim, De Colstoun, & McMurtrey Iii, 2000) |
| 61 | MCARI1 | Modified Chlorophyll Absorption in Reflectance Index 1 | $1.2(2.5(R_{790}-R_{670})-1.3(R_{790}-R_{550}))$ | (Haboudane, Miller, Pattey, Zarco-Tejada, & Strachan, 2004) |
| 62 | MCARI2 | Modified Chlorophyll Absorption in Reflectance Index 2 | $((R_{750} - R_{705}) - 0.2 * (R_{750} -R_{550})) * (R_{750}/R_{705})$ | (Haboudane et al., 2004) |
| 63 | MCARI2/OSAVI2 | | MCARI2/OSAVI2 | (C. Wu, Niu, Tang, & Huang, 2008) |
| 64 | MCARI/OSAVI | | MCARI2/OSAVI | (Daughtry et al., 2000) |
| 65 | mNDVI | Modified NDVI | $(R_{800} - R_{680})/(R_{800} + R_{680} – 2 * R_{445})$ | (Sims & Gamon, 2002) |
| 66 | mNDVI2 | Modified NDVI 2 | $(R_{750} - R_{705})/( R_{750} + R_{705} -2 * R_{445})$ | (Sims & Gamon, 2002) |
| 67 | MPRI | Modified Photochemical Reflectance Index | $(R_{515} - R_{530})/(R_{515} + R_{530})$ | (Hernández-Clemente, Navarro-Cerrillo, Suárez, Morales, & Zarco-Tejada, 2011) |
| 68 | mREIP | Modified Red-Edge Inflection Point | Modified REIP with inverted Gaussian fit on reflectance | (Miller, Hare, & Wu, 1990) |
| 69 | mSAVI | Modified Soil Adjusted Vegetation Index | $0.5 * (2 * R_{800} + 1- ((2 * R_{800} + 1)^2 – 8 * (R_{800} - R_{670}))^{0.5})$ | (Qi, Chehbouni, Huete, Kerr, & Sorooshian, 1994) |
| 70 | MSI | Moisture Stress Index | $R_{1600}/R_{817}$ | (Hunt Jr. & Rock, 1989) |

| | | | | |
|---|---|---|---|---|
| 71 | mSR | modified Simple Ratio | $(R_{800}-R_{445})/(R_{680}-R_{445})$ | (Sims & Gamon, 2002) |
| 72 | mSR2 | modified Simple Ratio 2 | $(R_{750}/R_{705})$ -1/$(R_{750}/R_{705})$ + 1)$^{0.5}$ | (Jing M Chen, 1996) |
| 73 | mSR3 | modified Simple Ratio 3 | $(R_{800}/(R_{670} - 1))/ (R_{800}/(R_{670} + 1))^{0.5}$ | (Jing M. Chen & Cihlar, 1996) |
| 74 | mSR705 | modified Simple Ratio 705 | $(R_{750} - R_{445})/(R_{705}-R_{445})$ | (Sims & Gamon, 2002) |
| 75 | MTCI | MERIS Terrestrial Chlorophyll Index | $(R_{754} - R_{709})/(R_{709} - R_{681})$ | (Dash & Curran, 2004) |
| 76 | mTVI | modified Triangular Vegetation Index | $1.2 * (1.2 * (R_{800} - R_{550}) - 2.5 * (R_{670} - R_{550}))$ | (Haboudane et al., 2004) |
| 77 | MVSR | Modified Vegetation Stress Ratio | $R_{723}/R_{700}$ | (White, Williams, & Barr, 2008) |
| 78 | NDVI4 | Narrow-Band Normalised Difference Vegetation Index | $(R_{850} - R_{680})/(R_{850} + R_{680})$ | (Cao et al., 2015) |
| 79 | NDLI | Normalized Difference Lignin Index | $(\log(1/ R_{1754}) - \log(1/ R_{1680}))/(\log(1/ R_{1754}) + \log(1/ R_{1680})$ | (Serrano, Peñuelas, & Ustin, 2002) |
| 80 | NDNI | Normalized Difference Nitrogen Index | $(\log(1/ R_{1510}) - \log(1/ R_{1680}))/(\log(1/ R_{1510}) + \log(1/ R_{1680}))$ | (Serrano et al., 2002) |
| 81 | NDVI | Normalized Difference Vegetation Index | $(R_{800} - R_{680})/(R_{800} + R_{680})$ | (Tucker, 1979) |
| 82 | NDVI2 | Normalized Difference Vegetation Index 2 | $(R_{750} - R_{705})/(R_{750} + R_{705})$ | (A. Gitelson & Merzlyak, 1994) |
| 83 | NDVI3 | Normalized Difference Vegetation Index 3 | $(R_{682} - R_{553})/(R_{682} + R_{553})$ | (Gandia et al., 2004) |
| 84 | NPCI | Normalized Pigment Chlorophyll Index | $(R_{680} - R_{430})/(R_{680} + R_{430})$ | (Peñuelas et al., 1994) |
| 85 | NPQI | Normalized Difference 415/435 Normalized Phaeophytinization Index | $(R_{415}-R_{435})/(R_{415}+R_{435})$ | (P. J. Zarco-Tejada, Miller, Noland, Mohammed, & Sampson, 2001) |
| 86 | NRI | Nitrogen Reflectance Index | $(R_{570} - R_{670})/(R_{570} + R_{670})$ | (Cao et al., 2015) |
| 87 | OSAVI | Optimized Soil Adjusted Vegetation Index | $(1 + 0.16) * (R_{800}-R_{670})/(R_{800} + R_{670} + 0.16)$ | (Rondeaux, Steven, & Baret, 1996) |
| 88 | OSAVI2 | Optimized Soil Adjusted Vegetation Index 2 | $(1 + 0.16) * (R_{750}-R_{705})/(R_{750} + R_{705} + 0.16)$ | (C. Wu et al., 2008) |
| 89 | RARS | Ratio Analysis of Reflectance Spectra | $R_{746}/R_{513}$ | (Chappelle, Kim, & McMurtrey, 1992) |
| 90 | PhRI | Physiological Reflectance Index | $(R_{550} - R_{531})/(R_{550} + R_{531})$ | (Cao et al., 2015) |
| 91 | PRI | Photochemical Reflectance Index | $(R_{531} - R_{570})/(R_{531} + R_{570})$ | (Gamon, Serrano, & Surfus, 1997) |

| | | | | |
|---|---|---|---|---|
| 92 | PRI2 | Photochemical Reflectance Index 2 | $(R_{570} - R_{539})/(R_{570} + R_{539})$ | (Iolanda Filella, Amaro, Araus, & Penuelas, 1996) |
| 93 | PRI*CI2 | | PRI*CI2 | (Garrity, Eitel, & Vierling, 2011) |
| 94 | PRI_norm | normalized PRI | $PRI * (-1)/(RDVI * R_{700}/R_{670})$ | (P. J. Zarco-Tejada et al., 2013) |
| 95 | PSND | Pigment specific normalised difference | $(R_{800} - R_{470})/(R_{800} + R_{470})$ | (Blackburn, 1998) |
| 96 | PSRI | Plant Senescence Reflectance Index | $(R_{678} - R_{500})/R_{750}$ | (Merzlyak, Gitelson, Chivkunova, & Rakitin, 1999) |
| 97 | PSSR | Pigment specific simple ratio | $R_{800}/R_{635}$ | (Blackburn, 1998) |
| 98 | PWI | Plant Water Index | $R_{900}/R_{970}$ | (Peñuelas, Pinol, Ogaya, & Filella, 1997) |
| 99 | RDVI | Renormalized Difference Vegetation Index | $(R_{800} - R_{670})/SQRT(R_{800} + R_{670})$ | (Roujean & Breon, 1995) |
| 100 | REP | Red-Edge Position | $700 + 40((R_{670} + R_{780})/2 – R_{700})/(R_{740} – R_{700}))$ | (Guyot, Baret, & Major, 1988) |
| 101 | REP_LE | - | Red-edge position through linear extrapolation | (Cho & Skidmore, 2006) |
| 102 | REP_Li | - | $R_{re} = (R_{670} + R_{780})/2$<br>$700 + 40 * ((R_{re} - R_{700})/(R_{740} - R_{700}))$ | (Guyot & Baret, 1988) |
| 103 | RGI | Red/Green Index | $R_{690}/R_{550}$ | (P. Zarco-Tejada et al., 2005) |
| 104 | SAVI | Soil Adjusted Vegetation Index | $(1 + L) * (R_{800} - R_{670})/(R_{800} + R_{670} + L)$ | (A. R. Huete, 1988) |
| 105 | SIPI | Structure Intensive Pigment Index | $(R_{800} - R_{445})/(R_{800} - R_{680})$ | (Peñuelas, Filella, Lloret, Muñoz, & Vilajeliu, 1995) |
| 106 | SIPI2 | Structure Intensive Pigment Index 2 | $(R_{800} - R_{440})/(R_{800} - R_{680})$ | (Pen Uelas, Filella, Lloret, MUN¯ OZ, & Vilajeliu, 1995) |
| 107 | SIPI3 | Structure Intensive Pigment Index 3 | $(R_{800} - R_{445})/(R_{800} - R_{650})$ | (Peñuelas, Baret, & Filella, 1995) |
| 108 | SPVI | Spectral Polygon Vegetation Index | $0.4 * (3.7 * (R_{800} - R_{670}) -1.2 * ((R_{530} - R_{670})^2)^{0.5}$ | (Vincini, Frazzi, & D'Alessio, 2006) |
| 109 | SR | Simple Ratio | $R_{800}/R_{680}$ | (Jordan, 1969) |
| 110 | SR1 | Simple Ratio 1 | $R_{750}/R_{700}$ | (A. A. Gitelson & Merzlyak, 1997) |

| | | | | |
|---|---|---|---|---|
| 111 | SR2 | Simple Ratio 2 | $R_{752}/R_{690}$ | (A. A. Gitelson & Merzlyak, 1997) |
| 112 | SR3 | Simple Ratio 3 | $R_{750}/ R_{550}$ | (A. A. Gitelson & Merzlyak, 1997) |
| 113 | SR4 | Simple Ratio 4 | $R_{700}/ R_{670}$ | (McMurtrey, Chappelle, Kim, Meisinger, & Corp, 1994) |
| 114 | SR5 | Simple Ratio 5 | $R_{675}/R_{700}$ | (Chappelle et al., 1992) |
| 115 | SR6 | Simple Ratio 6 | $R_{750}/R_{710}$ | (Zarco-Tejada & Miller, 1999) |
| 116 | SR7 | Simple Ratio 7 | $R_{440}/R_{690}$ | (Lichtenthaler et al., 1996) |
| 117 | SR705 | Simple Ratio 705 | $R_{750}/ R_{705}$ | (Castro-Esau, Sanchez-Azofeifa, & Rivard, 2006) |
| 118 | SR8 | Simple Ratio 8 | $R_{515}/R_{550}$ | (Hernández-Clemente, Navarro-Cerrillo, & Zarco-Tejada, 2012) |
| 119 | SR9 | Simple Ratio 9 | $R_{690}/R_{655}$ | (P. J. Zarco-Tejada, Pushnik, et al., 2003) |
| 120 | SR10 | Simple Ratio 10 | $R_{685}/R_{655}$ | (P. J. Zarco-Tejada, Pushnik, et al., 2003) |
| 121 | SRPI | Simple Ratio Pigment Index | $R_{430}/R_{680}$ | (J Peñuelas et al., 1995) |
| 122 | SRWI | Simple Ratio 850/1240 | $R_{850}/R_{1240}$ | (P. J. Zarco-Tejada, Rueda, & Ustin, 2003) |
| 123 | Sum_Dr1 | - | $\sum_{i=626}^{795} D1i$ | (Elvidge & Chen, 1995) |
| 124 | Sum_Dr2 | - | $\sum_{i=680}^{780} D1i$ | (I. Filella & Penuelas, 1994) |
| 125 | SWIR FI | Shortwave-infrared | $((R_{2133})^2 / R_{2225})*(R_{2209})^3$ | (Levin, Kidron, & Ben-Dor, 2007) |
| 126 | SWIR LI | Shortwave-infrared | $3.87*(R_{2210} - R_{2090}) - 27.51*(R_{2280} - R_{2090}) - 0.2$ | (Lobell, Asner, Law, & Treuhaft, 2001) |
| 127 | SWIR SI | Shortwave-infrared | $-41.59*(R_{2210} - R_{2090}) + 1.24*(R_{2280} - R_{2090}) - 0.64$ | (Lobell et al., 2001) |
| 128 | SWIR VI | Shortwave-infrared | $37.72*(R_{2210} - R_{2090}) + 26.27*(R_{2280} - R_{2090}) + 0.57$ | (Lobell et al., 2001) |

| | | | | |
|---|---|---|---|---|
| 129 | TCARI | Transformed Chlorophyll Absorbtion Ratio | $3 * ((R_{700} - R_{670}) - 0.2 * (R_{700} - R_{550}) * (R_{700}/R_{670}))$ | (Haboudane, Miller, Tremblay, Zarco-Tejada, & Dextraze, 2002) |
| 130 | TCARI2 | Transformed Chlorophyll Absorbtion Ratio 2 | $3 * ((R_{750} - R_{705}) - 0.2 * (R_{750} - R_{550}) * (R_{750}/R_{705}))$ | (C. Wu et al., 2008) |
| 131 | TCARI2/OSAVI2 | - | TCARI2/OSAVI2 | (C. Wu et al., 2008) |
| 132 | TCARI/OSAVI | - | TCARI/OSAVI | (Haboudane et al., 2002) |
| 133 | TGI | Triangular greenness index | $-0.5 * (190 * (R_{670} - R_{550}) – 120 * (R_{670} - R_{480}))$ | (Hunt et al., 2013) |
| 134 | TVI | Transformed Vegetation Index | $0.5 * (120 * (R_{750} - R_{550}) – 200 * (R_{670} - R_{550}))$ | (Broge & Leblanc, 2001) |
| 135 | Vog | Vogelmann indices | $R_{740}/R_{720}$ | (Vogelmann, Rock, & Moss, 1993) |
| 136 | Vog2 | Vogelmann indices 2 | $(R_{734} - R_{747})/(R_{715} + R_{726})$ | (Vogelmann et al., 1993) |
| 137 | Vog3 | Vogelmann indices 3 | $D_{715}/D_{705}$ | (Vogelmann et al., 1993) |
| 138 | Vog4 | Vogelmann indices 4 | $(R_{734} - R_{747})/(R_{715} + R_{720})$ | (Vogelmann et al., 1993) |
| 139 | VS | Vegetation Stress ratio | $R_{725}/ R_{702}$ | (White et al., 2008) |
| 140 | WI | Water Index | $R_{900}/R_{970}$ | (Peñuelas, Filella, Biel, Serrano, & Save, 1993) |

$R_x$ - reflectance at wavelength x nm.

$D_x$ - derivative of the reflectance spectrum at wavelength x nm.

- No original index name or abbreviation found

## References


Agapiou, A., Hadjimitsis, D. G., & Alexakis, D. D. (2012). Evaluation of broadband and narrowband vegetation indices for the identification of archaeological crop marks. *Remote sensing, 4*(12), 3892-3919.

Apan, A., Held, A., Phinn, S., & Markley, J. (2004). Detecting sugarcane 'orange rust' disease using EO-1 Hyperion hyperspectral imagery. *International Journal of Remote Sensing, 25*(2), 489-498. doi:10.1080/01431160310001618031

Ashourloo, D., Mobasheri, M., & Huete, A. (2014). Developing Two Spectral Disease Indices for Detection of Wheat Leaf Rust (Pucciniatriticina). *Remote Sensing, 6*(6), 4723-4740. doi:10.3390/rs6064723

Blackburn, G. A. (1998). Spectral indices for estimating photosynthetic pigment concentrations: A test using senescent tree leaves. *International Journal of Remote Sensing, 19*(4), 657-675. doi:10.1080/014311698215919

Boochs, F., Kupfer, G., Dockter, K., & Kühbauch, W. (1990). Shape of the red edge as vitality indicator for plants. *International Journal of Remote Sensing, 11*(10), 1741-1753. doi:10.1080/01431169008955127

Broge, N. H., & Leblanc, E. (2001). Comparing prediction power and stability of broadband and hyperspectral vegetation indices for estimation of green leaf area index and canopy chlorophyll density. *Remote Sensing of Environment, 76*(2), 156-172. doi:10.1016/s0034-4257(00)00197-8

Cao, X., Luo, Y., Zhou, Y., Fan, J., Xu, X., West, J. S., . . . Cheng, D. (2015). Detection of Powdery Mildew in Two Winter Wheat Plant Densities and Prediction of Grain Yield Using Canopy Hyperspectral Reflectance. *PLoS ONE, 10*(3). doi:10.1371/journal.pone.0121462

Carter, G. A. (1994). Ratios of leaf reflectances in narrow wavebands as indicators of plant stress. *International Journal of Remote Sensing, 15*(3), 697-703. doi:10.1080/01431169408954109

Castro-Esau, K., Sanchez-Azofeifa, G., & Rivard, B. (2006). Comparison of spectral indices obtained using multiple spectroradiometers. *Remote Sensing of Environment, 103*(3), 276-288. doi:10.1016/j.rse.2005.01.019

Chappelle, E. W., Kim, M. S., & McMurtrey, J. E. (1992). Ratio analysis of reflectance spectra (RARS): An algorithm for the remote estimation of the concentrations of chlorophyll A, chlorophyll B, and carotenoids in soybean leaves. *Remote Sensing of Environment, 39*(3), 239-247. doi:10.1016/0034-4257(92)90089-3

Chen, J. M. (1996). Evaluation of vegetation indices and a modified simple ratio for boreal applications. *Canadian Journal of Remote Sensing, 22*(3), 229-242.

Chen, J. M., & Cihlar, J. (1996). Retrieving leaf area index of boreal conifer forests using Landsat TM images. *Remote Sensing of Environment, 55*(2), 153-162. doi:10.1016/0034-4257(95)00195-6

Cho, M. A., & Skidmore, A. K. (2006). A new technique for extracting the red edge position from hyperspectral data: The linear extrapolation method. *Remote Sensing of Environment, 101*(2), 181-193. doi:10.1016/j.rse.2005.12.011

Dash, J., & Curran, P. J. (2004). The MERIS terrestrial chlorophyll index. *International Journal of Remote Sensing, 25*(23), 5403-5413. doi:10.1080/0143116042000274015

Datt, B. (1998). Remote Sensing of Chlorophyll a, Chlorophyll b, Chlorophyll a+b, and Total Carotenoid Content in Eucalyptus Leaves. *Remote Sensing of Environment, 66*(2), 111-121. doi:10.1016/s0034-4257(98)00046-7

Datt, B. (1999). Visible/near infrared reflectance and chlorophyll content in Eucalyptus leaves. *International Journal of Remote Sensing, 20*(14), 2741-2759. doi:10.1080/014311699211778

Daughtry, C., Walthall, C., Kim, M., De Colstoun, E. B., & McMurtrey Iii, J. (2000). Estimating corn leaf chlorophyll concentration from leaf and canopy reflectance. *Remote Sensing of Environment, 74*(2), 229-239. doi:10.1016/S0034-4257(00)00113-9

Elvidge, C. D., & Chen, Z. (1995). Comparison of broad-band and narrow-band red and near-infrared vegetation indices. *Remote Sensing of Environment, 54*(1), 38-48. doi:10.1016/0034-4257(95)00132-k

Filella, I., Amaro, T., Araus, J. L., & Penuelas, J. (1996). Relationship between photosynthetic radiation-use efficiency of barley canopies and the photochemical reflectance index (PRI). *Physiologia Plantarum, 96*(2), 211-216. doi:10.1111/j.1399-3054.1996.tb00204.x

Filella, I., & Penuelas, J. (1994). The red edge position and shape as indicators of plant chlorophyll content, biomass and hydric status. *International Journal of Remote Sensing, 15*(7), 1459-1470. doi:10.1080/01431169408954177

Galvão, L. S., Formaggio, A. R., & Tisot, D. A. (2005). Discrimination of sugarcane varieties in Southeastern Brazil with EO-1 Hyperion data. *Remote Sensing of Environment, 94*(4), 523-534. doi:10.1016/j.rse.2004.11.012

Gamon, J. A., Serrano, L., & Surfus, J. S. (1997). The photochemical reflectance index: an optical indicator of photosynthetic radiation use efficiency across species, functional types, and nutrient levels. *Oecologia, 112*(4), 492-501. doi:10.1007/s004420050337

Gandia, S., Fernández, G., García, J., & Moreno, J. (2004). Retrieval of vegetation biophysical variables from CHRIS/PROBA data in the SPARC campaign. *Esa Sp, 578*, 40-48.

Garrity, S. R., Eitel, J. U. H., & Vierling, L. A. (2011). Disentangling the relationships between plant pigments and the photochemical reflectance index reveals a new approach for remote estimation of carotenoid content. *Remote Sensing of Environment, 115*(2), 628-635. doi:10.1016/j.rse.2010.10.007

Gitelson, A., & Merzlyak, M. N. (1994). Quantitative estimation of chlorophyll-a using reflectance spectra: Experiments with autumn chestnut and maple leaves. *Journal of Photochemistry and Photobiology B: Biology, 22*(3), 247-252. doi:10.1016/1011-1344(93)06963-4

Gitelson, A. A., Buschmann, C., & Lichtenthaler, H. K. (1999). The Chlorophyll Fluorescence Ratio F735/F700 as an Accurate Measure of the Chlorophyll Content in Plants. *Remote Sensing of Environment, 69*(3), 296-302. doi:10.1016/s0034-4257(99)00023-1

Gitelson, A. A., Gritz, Y., & Merzlyak, M. N. (2003). Relationships between leaf chlorophyll content and spectral reflectance and algorithms for non-destructive chlorophyll assessment in higher plant leaves. *Journal of Plant Physiology, 160*(3), 271-282. doi:10.1078/0176-1617-00887

Gitelson, A. A., Kaufman, Y. J., & Merzlyak, M. N. (1996). Use of a green channel in remote sensing of global vegetation from EOS-MODIS. *Remote Sensing of Environment, 58*(3), 289-298. doi:10.1016/s0034-4257(96)00072-7

Gitelson, A. A., & Merzlyak, M. N. (1997). Remote estimation of chlorophyll content in higher plant leaves. *International Journal of Remote Sensing, 18*(12), 2691-2697. doi:10.1080/014311697217558

Gitelson, A. A., & Merzlyak, M. N. (1998). Remote sensing of chlorophyll concentration in higher plant leaves. *Advances in Space Research, 22*(5), 689-692. doi:10.1016/s0273-1177(97)01133-2

Gitelson, A. A., Merzlyak, M. N., & Chivkunova, O. B. (2001). Optical properties and nondestructive estimation of anthocyanin content in plant leaves. *Photochemistry and Photobiology, 74*(1), 38-45. doi:10.1562/0031-8655(2001)0740038OPANEO2.0.CO2

Gitelson, A. A., Zur, Y., Chivkunova, O. B., & Merzlyak, M. N. (2002). Assessing carotenoid content in plant leaves with reflectance spectroscopy. *Photochemistry and Photobiology, 75*(3), 272-281. doi:10.1562/0031-8655(2002)0750272ACCIPL2.0.CO2

Guyot, G., & Baret, F. (1988). *Utilisation de la haute resolution spectrale pour suivre l'etat des couverts vegetaux.* Paper presented at the Spectral Signatures of Objects in Remote Sensing.

Guyot, G., Baret, F., & Major, D. (1988). High spectral resolution: Determination of spectral shifts between the red and the near infrared. *International Archives of Photogrammetry and Remote Sensing, 11*(750-760), 750-760.

Haboudane, D., Miller, J. R., Pattey, E., Zarco-Tejada, P. J., & Strachan, I. B. (2004). Hyperspectral vegetation indices and novel algorithms for predicting green LAI of crop canopies: Modeling and validation in the context of precision agriculture. *Remote Sensing of Environment, 90*(3), 337-352. doi:10.1016/j.rse.2003.12.013

Haboudane, D., Miller, J. R., Tremblay, N., Zarco-Tejada, P. J., & Dextraze, L. (2002). Integrated narrow-band vegetation indices for prediction of crop chlorophyll content for application to precision agriculture. *Remote Sensing of Environment, 81*(2-3), 416-426. doi:10.1016/s0034-4257(02)00018-4

Hernández-Clemente, R., Navarro-Cerrillo, R. M., Suárez, L., Morales, F., & Zarco-Tejada, P. J. (2011). Assessing structural effects on PRI for stress detection in conifer forests. *Remote Sensing of Environment, 115*(9), 2360-2375. doi:10.1016/j.rse.2011.04.036

Hernández-Clemente, R., Navarro-Cerrillo, R. M., & Zarco-Tejada, P. J. (2012). Carotenoid content estimation in a heterogeneous conifer forest using narrow-band indices and PROSPECT+DART simulations. *Remote Sensing of Environment, 127*, 298-315. doi:10.1016/j.rse.2012.09.014

Huete, A. R. (1988). A soil-adjusted vegetation index (SAVI). *Remote Sensing of Environment, 25*(3), 295-309. doi:10.1016/0034-4257(88)90106-x

Huete, A. R., Liu, H. Q., Batchily, K., & Van Leeuwen, W. (1997). A comparison of vegetation indices over a global set of TM images for EOS-MODIS. *Remote Sensing of Environment, 59*(3), 440-451. doi:10.1016/s0034-4257(96)00112-5

Hunt, E. R., Doraiswamy, P. C., McMurtrey, J. E., Daughtry, C. S. T., Perry, E. M., & Akhmedov, B. (2013). A visible band index for remote sensing leaf chlorophyll content at the canopy scale. *International Journal of Applied Earth Observation and Geoinformation, 21*, 103-112. doi:10.1016/j.jag.2012.07.020

Hunt Jr., E., & Rock, B. (1989). Detection of changes in leaf water content using Near- and Middle-Infrared reflectances☆. *Remote Sensing of Environment, 30*(1), 43-54. doi:10.1016/0034-4257(89)90046-1

Jordan, C. F. (1969). Derivation of Leaf-Area Index from Quality of Light on the Forest Floor. *Ecology, 50*(4), 663-666. doi:10.2307/1936256

Kim, M. S., Daughtry, C., Chappelle, E., McMurtrey, J., & Walthall, C. (1994). The use of high spectral resolution bands for estimating absorbed photosynthetically active radiation (A par). *CNES, Proceedings of 6th International Symposium on Physical Measurements and Signatures in Remote Sensing*, 299-306.

le Maire, G., François, C., & Dufrêne, E. (2004). Towards universal broad leaf chlorophyll indices using PROSPECT simulated database and hyperspectral reflectance measurements. *Remote Sensing of Environment, 89*(1), 1-28. doi:10.1016/j.rse.2003.09.004

le Maire, G., Francois, C., Soudani, K., Berveiller, D., Pontailler, J., Breda, N., . . . Dufrene, E. (2008). Calibration and validation of hyperspectral indices for the estimation of broadleaved forest leaf chlorophyll content, leaf mass per area, leaf area index and leaf canopy biomass. *Remote Sensing of Environment, 112*(10), 3846-3864. doi:10.1016/j.rse.2008.06.005

Lehnert, L., Meyer, H., & Bendix, J. (2016). hsdar: Manage, analyse and simulate hyperspectral data in R. *R Package Version 0.4, 1*.

Levin, N., Kidron, G. J., & Ben-Dor, E. (2007). Surface properties of stabilizing coastal dunes: combining spectral and field analyses. *Sedimentology, 54*(4), 771-788. doi:10.1111/j.1365-3091.2007.00859.x

Lichtenthaler, H. K., Lang, M., Sowinska, M., Heisel, F., & Miehé, J. A. (1996). Detection of Vegetation Stress Via a New High Resolution Fluorescence Imaging System. *Journal of Plant Physiology, 148*(5), 599-612. doi:10.1016/s0176-1617(96)80081-2

Lobell, D. B., Asner, G. P., Law, B. E., & Treuhaft, R. N. (2001). Subpixel canopy cover estimation of coniferous forests in Oregon using SWIR imaging spectrometry. *Journal of Geophysical Research: Atmospheres, 106*(D6), 5151-5160. doi:10.1029/2000jd900739

Maccioni, A., Agati, G., & Mazzinghi, P. (2001). New vegetation indices for remote measurement of chlorophylls based on leaf directional reflectance spectra. *J Photochem Photobiol B, 61*(1-2), 52-61. doi:10.1016/S1011-1344(01)00145-2

Main, R., Cho, M. A., Mathieu, R., O'Kennedy, M. M., Ramoelo, A., & Koch, S. (2011). An investigation into robust spectral indices for leaf chlorophyll estimation. *ISPRS Journal of Photogrammetry and Remote Sensing, 66*(6), 751-761. doi:10.1016/j.isprsjprs.2011.08.001

McMurtrey, J. E., Chappelle, E. W., Kim, M. S., Meisinger, J. J., & Corp, L. A. (1994). Distinguishing nitrogen fertilization levels in field corn (Zea mays L.) with actively induced fluorescence and passive reflectance measurements. *Remote Sensing of Environment, 47*(1), 36-44. doi:10.1016/0034-4257(94)90125-2

Merzlyak, M. N., Gitelson, A. A., Chivkunova, O. B., & Rakitin, V. Y. U. (1999). Non-destructive optical detection of pigment changes during leaf senescence and fruit ripening. *Physiologia Plantarum, 106*(1), 135-141. doi:10.1034/j.1399-3054.1999.106119.x

Miller, J. R., Hare, E. W., & Wu, J. (1990). Quantitative characterization of the vegetation red edge reflectance 1. An inverted-Gaussian reflectance model. *International Journal of Remote Sensing, 11*(10), 1755-1773. doi:10.1080/01431169008955128

Nagler, P. L., Inoue, Y., Glenn, E. P., Russ, A. L., & Daughtry, C. S. T. (2003). Cellulose absorption index (CAI) to quantify mixed soil–plant litter scenes. *Remote Sensing of Environment, 87*(2-3), 310-325. doi:10.1016/j.rse.2003.06.001

Odilbekov, F., Armonienė, R., Henriksson, T., & Chawade, A. (2018). Proximal phenotyping and machine learning methods to identify Septoria tritici blotch disease symptoms in wheat. *Front Plant Sci*. doi:10.3389/fpls.2018.00685

Oppelt, N., & Mauser, W. (2004). Hyperspectral monitoring of physiological parameters of wheat during a vegetation period using AVIS data. *International Journal of Remote Sensing, 25*(1), 145-159. doi:10.1080/0143116031000115300

Pen Uelas, J., Filella, I., Lloret, P., MUN¯ OZ, F., & Vilajeliu, M. (1995). Reflectance assessment of mite effects on apple trees. *International Journal of Remote Sensing, 16*(14), 2727-2733.

Peñuelas, J., Baret, F., & Filella, I. (1995). Semi-empirical indices to assess carotenoids/chlorophyll a ratio from leaf spectral reflectance. *Photosynthetica, 31*(2), 221-230.

Peñuelas, J., Filella, I., Biel, C., Serrano, L., & Save, R. (1993). The reflectance at the 950–970 nm region as an indicator of plant water status. *International Journal of Remote Sensing, 14*(10), 1887-1905. doi:10.1080/01431169308954010

Peñuelas, J., Filella, I., Lloret, P., Muñoz, F., & Vilajeliu, M. (1995). Reflectance assessment of mite effects on apple trees. *International Journal of Remote Sensing, 16*(14), 2727-2733. doi:10.1080/01431169508954588

Peñuelas, J., Gamon, J. A., Fredeen, A. L., Merino, J., & Field, C. B. (1994). Reflectance indices associated with physiological changes in nitrogen- and water-limited sunflower leaves. *Remote Sensing of Environment*, *48*(2), 135-146. doi:10.1016/0034-4257(94)90136-8

Peñuelas, J., Pinol, J., Ogaya, R., & Filella, I. (1997). Estimation of plant water concentration by the reflectance Water Index WI (R900/R970). *International Journal of Remote Sensing, 18*(13), 2869-2875. doi:10.1080/014311697217396

Qi, J., Chehbouni, A., Huete, A. R., Kerr, Y. H., & Sorooshian, S. (1994). A modified soil adjusted vegetation index. *Remote Sensing of Environment, 48*(2), 119-126. doi:10.1016/0034-4257(94)90134-1

Rondeaux, G., Steven, M., & Baret, F. (1996). Optimization of soil-adjusted vegetation indices. *Remote Sensing of Environment, 55*(2), 95-107. doi:10.1016/0034-4257(95)00186-7

Roujean, J.-L., & Breon, F.-M. (1995). Estimating PAR absorbed by vegetation from bidirectional reflectance measurements. *Remote Sensing of Environment, 51*(3), 375-384. doi:10.1016/0034-4257(94)00114-3

Serrano, L., Peñuelas, J., & Ustin, S. L. (2002). Remote sensing of nitrogen and lignin in Mediterranean vegetation from AVIRIS data. *Remote Sensing of Environment, 81*(2-3), 355-364. doi:10.1016/s0034-4257(02)00011-1

Sims, D. A., & Gamon, J. A. (2002). Relationships between leaf pigment content and spectral reflectance across a wide range of species, leaf structures and developmental stages. *Remote Sensing of Environment*, *81*(2-3), 337-354. doi:10.1016/s0034-4257(02)00010-x

Smith, R. C. G., Adams, J., Stephens, D. J., & Hick, P. T. (1995). Forecasting wheat yield in a Mediterranean-type environment from the NOAA satellite. *Australian Journal of Agricultural Research, 46*(1), 113-125. doi:10.1071/ar9950113

Tucker, C. J. (1979). Red and photographic infrared linear combinations for monitoring vegetation. *Remote Sensing of Environment*, *8*(2), 127-150. doi:10.1016/0034-4257(79)90013-0

Vincini, M., Frazzi, E., & D'Alessio, P. (2006). *Angular dependence of maize and sugar beet VIs from directional CHRIS/Proba data.* Paper presented at the Proc. 4th ESA CHRIS PROBA Workshop.

Vogelmann, J. E., Rock, B. N., & Moss, D. M. (1993). Red edge spectral measurements from sugar maple leaves. *International Journal of Remote Sensing, 14*(8), 1563-1575. doi:10.1080/01431169308953986

White, D., Williams, M., & Barr, S. (2008). Detecting sub-surface soil disturbance using hyperspectral first derivative band rations of associated vegetation stress. *Int. Arch. Photogramm. Remote Sens. Spat. Inf. Sci, 37*, 243-248.

Wu, C., Niu, Z., Tang, Q., & Huang, W. (2008). Estimating chlorophyll content from hyperspectral vegetation indices: Modeling and validation. *Agricultural and Forest Meteorology, 148*(8-9), 1230-1241. doi:10.1016/j.agrformet.2008.03.005

Wu, W. (2014). The Generalized Difference Vegetation Index (GDVI) for Dryland Characterization. *Remote Sensing, 6*(2), 1211-1233. doi:10.3390/rs6021211

Zarco-Tejada, P., Berjon, A., Lopezlozano, R., Miller, J., Martin, P., Cachorro, V., . . . Defrutos, A. (2005). Assessing vineyard condition with hyperspectral indices: Leaf and canopy reflectance simulation in a row-structured discontinuous canopy. *Remote Sensing of Environment, 99*(3), 271-287. doi:10.1016/j.rse.2005.09.002

Zarco-Tejada, P. J., González-Dugo, V., Williams, L. E., Suárez, L., Berni, J. A. J., Goldhamer, D., & Fereres, E. (2013). A PRI-based water stress index combining structural and chlorophyll effects: Assessment using diurnal narrow-band airborne imagery and the CWSI thermal index. *Remote Sensing of Environment, 138*, 38-50. doi:10.1016/j.rse.2013.07.024

Zarco-Tejada, P. J., Miller, J. R., Noland, T. L., Mohammed, G. H., & Sampson, P. H. (2001). Scaling-up and model inversion methods with narrowband optical indices for chlorophyll content estimation in closed forest canopies with hyperspectral data. *IEEE Transactions on Geoscience and Remote Sensing, 39*(7), 1491-1507. doi:10.1109/36.934080

Zarco-Tejada, P. J., Pushnik, J. C., Dobrowski, S., & Ustin, S. L. (2003). Steady-state chlorophyll a fluorescence detection from canopy derivative reflectance and double-peak red-edge effects. *Remote Sensing of Environment, 84*(2), 283-294. doi:10.1016/s0034-4257(02)00113-x

Zarco-Tejada, P. J., Rueda, C. A., & Ustin, S. L. (2003). Water content estimation in vegetation with MODIS reflectance data and model inversion methods. *Remote Sensing of Environment, 85*(1), 109-124. doi:10.1016/s0034-4257(02)00197-9

Zarco-Tejada, P. J., & Miller, J. R. (1999). Land cover mapping at BOREAS using red edge spectral parameters from CASI imagery. *Journal of Geophysical Research: Atmospheres, 104*(D22), 27921-27933.
